# Multiple-antigen ELISA for melioidosis - a novel approach to the improved serodiagnosis of melioidosis

**DOI:** 10.1186/1471-2334-13-165

**Published:** 2013-04-04

**Authors:** Yuka Hara, Chui-Yoke Chin, Rahmah Mohamed, Savithri D Puthucheary, Sheila Nathan

**Affiliations:** 1 Malaysia Genome Institute, Jalan Bangi, Kajang, Selangor D.E, 43000, Malaysia; 2 School of Biosciences and Biotechnology, Faculty of Science and Technology, Universiti Kebangsaan Malaysia, Bangi, Selangor D.E, 43600, Malaysia; 3 Medical Education, Research and Evaluation Department, Duke-NUS Graduate Medical School Singapore, 8 College Road, Singapore, 169857, Singapore; 4 Current affiliation: Emory Vaccine Center, Emory University, Atlanta, GA, USA

**Keywords:** Melioidosis, Serodiagnosis, ELISA, TssD-5 and Omp3

## Abstract

**Background:**

*Burkholderia pseudomallei*, the causative agent of melioidosis, is endemic to Southeast Asia and northern Australia. Clinical manifestations of disease are diverse, ranging from chronic infection to acute septicaemia. The current gold standard of diagnosis involves bacterial culture and identification which is time consuming and often too late for early medical intervention. Hence, rapid diagnosis of melioidosis is crucial for the successful management of melioidosis.

**Methods:**

The study evaluated 4 purified *B. pseudomallei* recombinant proteins (TssD-5, Omp3, smBpF4 and Omp85) as potential diagnostic agents for melioidosis. A total of 68 sera samples from Malaysian melioidosis patients were screened for the presence of specific antibodies towards these proteins using enzyme-linked immunosorbent assay (ELISA). Sera from patients with various bacterial and viral infections but negative for *B. pseudomallei*, as well as sera from healthy individuals, were also included as non-melioidosis controls. The Mann Whitney test was performed to compare the statistical differences between melioidosis patients and the non-melioidosis controls.

**Results:**

TssD-5 demonstrated the highest sensitivity of 71% followed by Omp3 (59%), smBpF4 (41%) and Omp85 (19%). All 4 antigens showed equally high specificity (89-96%). A cocktail of all 4 antigens resulted in slightly reduced sensitivity of 65% but improved specificity (99%). Multiple-antigen ELISA provided improved sensitivity of 88.2% whilst retaining good specificity (96%). There was minimal reactivity with sera from healthy individuals proposing the utility of these antigens to demarcate diseased from non-symptomatic individuals in an endemic country.

**Conclusions:**

TssD-5 demonstrated high detection sensitivity and specificity and the results were obtained within a few hours compared to time consuming culture and IFAT methods commonly used in a clinical setting. The use of multiple-antigens resulted in improved sensitivity (88.2%) whilst maintaining superior specificity. These data highlight the use of TssD-5 and other recombinant antigens tested in this study as potential serodiagnostic agents for melioidosis.

## Background

Melioidosis is a severe community-acquired infectious disease caused by the Gram negative bacillus *Burkholderia pseudomallei*. The bacterium is commonly found in soil and water in Southeast Asia and northern Australia; however, increasing cases of melioidosis have been reported in other tropical regions and the bacterium is now believed to present a serious global threat [1,2]. Clinical manifestations of melioidosis are extremely diverse and vary from acute sepsis to chronic localised pathology to latent infections which can reactivate decades later [3,4]. In all series, the lung was the most commonly affected organ, either presenting with cough and fever resulting from a primary lung abscess or pneumonia, or secondary to septicaemic spread [5]. The overall mortality rate in individuals infected with *B. pseudomallei* range from 30-70%, depending on whether the patient is septicaemic or not [5]. In northeast Thailand the mortality rate is reported to be 50% (35% in children) and 19% in Australia [3,6].

Melioidosis is treatable if detected early, however, early medical intervention is hindered by two major factors; firstly, the definitive diagnosis involves bacterial culture and identification which is time consuming and often leads to a critical delay in antibiotic therapy and successful management of the acute form of the disease [7]. Secondly, *B. pseudomallei* is resistant to diverse groups of antimicrobials including third generation cephalosporins whilst quinolones and aminoglycosides have no reliable effect [8,9]. Hence, a definitive and rapid diagnosis is critical and vital prior to the administration of ceftazidime or carbapenems as these antibiotics are generally not used as empirical treatment for septicaemia in endemic regions. Culturing of bacteria from clinical samples represents the diagnostic gold standard for melioidosis [3,10]. Whilst it is simple, reliable and economical, the long duration required to reach a definitive diagnosis is a major drawback. The most commonly used serological method, the indirect hemagglutination test (IHA), is poorly standardised worldwide. Moreover, IHA has limited clinical value in regions of endemicity due to the high background antibody titres in healthy individuals, most likely the result of repeated environmental exposure to *B. pseudomallei*[11]. In addition, IHA uses crude whole-cell preparation or bacterial lysates which may potentially lead to high false-positive results. A critical limitation of this assay is the lack of standardisation between laboratories with respect to the antigens used, the antigens remain poorly characterised and are likely to be variable between isolates [12]. The indirect immune-fluorescence antibody test (IFAT) using whole *B. pseudomallei* cells as antigen was found to be sensitive and superior to IHA and requires only a day to obtain the results [13]. The only drawback is that IFAT requires a fluorescence microscope and skilled personnel which might not be readily available in rural endemic regions of Southeast Asia. Enzyme-linked immunosorbent assay (ELISA) and related serodiagnostic strategies are being considered more favourably as rapid and reliable tools for definitive diagnosis of melioidosis [14-16]. Various antigen preparations such as crude and purified exopolysaccharide (EPS) and lipopolysaccharide (LPS) as well as recombinant flagellin and Bip proteins have been reported as potential diagnostic antigens in an ELISA format, however, sensitive and reliable antigens are yet to be identified.

We have previously reported the characterisation of several immunogenic *B. pseudomallei* recombinant proteins including outer membrane protein A (Omp3), Omp85 and serine protease MprA (smBpF4) [17-19]. Recently, Chieng et al. [20] described the elevated induction levels of *B. pseudomallei* type VI secretion system HCP protein (TssD-5) over a 6 hr infection period in human macrophages and preliminary analysis of the antigenicity of this protein has indicated strong binding to antibodies in a small cohort of melioidosis-confirmed patient sera (Chieng et al. *in preparation*). All four recombinant proteins are unique to *Burkholderia spp*. and interestingly, TssD-5 is highly conserved in *B. pseudomallei* only whilst the other recombinant proteins are conserved in *B. pseudomallei* as well as in *Burkholderia spp*. such as *B. mallei* and *B. thailandensis*. Thus, all the selected proteins should demonstrate minimal cross reactivity with sera from non-melioidosis individuals presenting with other bacterial infections. Concomitantly, these 4 recombinant proteins were strongly reactive with melioidosis patients’ or *B. pseudomallei* infected animal sera [17-19]. As these *B. pseudomallei* recombinant proteins were patient sero-reactive proteins, we attempt to evaluate their potential as suitable antigen(s) for diagnosis of melioidosis by an ELISA-based screen, a rapid diagnostic method to replace the conventional gold standard which involves bacterial culture and is time consuming.

## Methods

### Bacterial strains and plasmids

The *B. pseudomallei* human isolate (strain D286) was obtained from the Pathogen Laboratory, School of Biosciences and Biotechnology, Faculty of Science and Technology, Universiti Kebangsaan Malaysia. This strain was isolated from a patient with clinical manifestations of melioidosis at the Kuala Lumpur Hospital and previously characterised based on biochemical tests as well as by 16S rRNA sequencing [21]. The recombinant *B. pseudomallei* constructs used in this study are summarised in Table 1.

**Table 1:** **Recombinant *B. pseudomallei *constructs used in this study**

**Recombinant constructs**	**Gene ID**	**Expressed protein molecular weight (kDa)**	**References**
Serine protease MprA, smBpF4	BPSS1993	55	[23]
Outer membrane protein A, Omp3	BPSL2522	27	[17]
Outer membrane protein, Omp85	BPSL2151	89	[18]
Type VI secretion system HCP protein, TssD-5	BPSS1498	22.9	Chieng et al. *in preparation*

### Human sera samples

Collection and use of melioidosis sera samples from patients was approved by the Medical Ethics Committee, University Malaya Medical Centre, Malaysia (Medical Ethics Committee ref. no. 260.1). The patient samples were taken as part of standard patient care. A total of 68 sera samples from culture-confirmed [22] or indirect IFAT-confirmed [13] melioidosis patients from Malaysia presenting with acute and chronic disease manifestations were screened. The detection of total antibodies to melioidosis by IFAT was performed as previously described [13]. A cut-off value of 1:80 was used to differentiate between true infections from background titres due to basal antibody levels in endemic areas [13]. A summary of the ethnic distribution of the sampled individuals is provided in Table 2.

**Table 2:** Demographics of 68 cases of melioidosis

**Characteristics**			**Study sample**	
		**N**	**%**	
**Total**		68	
**Gender**	Male	53	(77.9)
	Female	12	(17.6)
	Unknown	3	(4.4)
**Ethnic origin**	Malay	48	(70.6)
	Chinese	9	(13.2)
	Indian	9	(13.2)
	Unknown	2	(2.9)
**Age Category**	<30	6	(8.8)
	30-50	30	(44.1)
	51-70	12	(17.6)
	>70	2	(2.9)
	Unknown	18	(26.5)

Sera (n = 29) from individuals presenting with various bacterial and viral infections (*Legionella* (n = 6), *Mycoplasma* (n = 5), *Leptospira* (n = 4), *Chlamydia* (n = 4), endemic typhus (n = 3), typhoid (n = 3), scrub typhus (n = 2), dengue (n = 1) and Chikugunya (n = 1)) but confirmed *B. pseudomallei*-negative were used as disease controls. Control sera from healthy and confirmed HIV-negative individuals (n = 61) were generously provided by Clinipath Malaysia Sdn. Bhd.

### Preparation of *B. pseudomallei* lysate

*B. pseudomallei* D286 lysate was prepared as previously described with minor modifications [21]. Briefly, *B. pseudomallei* D286 was grown in brain heart infusion (BHI) broth overnight at 37°C and the overnight culture was centrifuged at 4,000 × *g* for 20 min at 4°C. The bacterial pellets obtained were washed twice with PBS. The cell suspension was heat-killed at 80°C for 1 hr and subsequently disrupted by sonication for 15 min on ice (Vibracell, Sonics and Materials). The lysate was then quantified by the Bicinchoninic Acid assay.

### Over-expression and purification of recombinant *B. pseudomallei* proteins

Over-expression and purification of recombinant *B. pseudomallei* Omp3 [17], Omp85 [18] and smBpF4 proteins [23] were performed as previously described. For TssD-5, the corresponding coding region of *tssD-5* was amplified by polymerase chain reaction from the *B. pseudomallei* D286 genomic DNA as previously described [17]. The primer pairs used were TssD-5_F (CACCATGCTGGCCGGAATATA) and TssD-5_R (TCAGCCATTCGTCCAGTTTG) designed based on the *B. pseudomallei* K96243 annotated ORF. Thirty cycles of amplification were performed according to the manufacturer’s recommended protocol using Expand High Fidelity PCR system (Roche) with an annealing temperature of 53.2°C. The purified PCR product was cloned into pET 200/D-TOPO® according to the manufacturer’s recommendation (Invitrogen). Gene orientation and sequences were verified by DNA sequencing. Recombinant *B. pseudomallei* TssD-5 proteins were expressed as soluble proteins in *Escherichia coli* BL21 (DE3) Star at 37°C with 1 mM isopropyl-β-D-thiogalactoside (IPTG) for 5 hrs. Expression cultures were harvested and bacterial pellets were re-suspended in BugBuster® Protein Extraction Reagent (Novagen). The suspension was incubated at room temperature for 20 min with rotation, then centrifuged at 12,000 × *g* for 30 min at 4°C and the supernatant was collected. The expressed soluble recombinant TssD-5 protein was purified under native conditions through the HisTrap HP 1 ml column on the AKTAPurifier system (GE, Healthcare) according to the manufacturer’s recommended protocol (Chieng et al. *in preparation*).

### ELISA

ELISA was performed in CELLSTAR 96 Well Polystyrene Flat Bottom Microplates (Greiner Bio One) as previously described with minor modifications [17,18]. Wells were coated overnight at 4°C with 0.25 μg per well of purified recombinant *B. pseudomallei* proteins or *B. pseudomallei* D286 lysate in coating bicarbonate buffer (0.1 M sodium bicarbonate, pH 9.6) in a 100 μl final volume. For the antigen cocktail, 4 recombinant proteins (0.25 μg each) were mixed in coating bicarbonate buffer (0.1 M sodium bicarbonate, pH 9.6) in a 100 μl final volume and coated in individual wells. All the antigens were initially denatured at 80°C for 10 min before coating of wells. Following overnight incubation, wells were washed 5 times with distilled water and blocked with blocking buffer [5% (w/v) skim milk in PBS] for 1 hr at 37°C. After 5 washes with distilled water, triplicate wells were incubated at 37°C for 1 hr with various human sera samples diluted (1:1600) in blocking buffer to a volume of 100 μl. Following incubation, wells were washed 5 times with distilled water and incubated at 37°C for 1 hr with 100 μl of rabbit anti-human IgG conjugated with peroxidase (1:10,000 diluted in blocking buffer, Sigma-Aldrich). Subsequently, 100 μl ABTS:peroxidase B (1:1; KPL) was added as substrate. The reaction was left to develop for 30 min at room temperature. The absorbance was measured at 405 nm with an automated Sunrise ELISA reader (Tecan). The mean and standard deviations of repeated measurements were calculated for each tested specimen. The cut-off point was determined as the mean of the 61 healthy controls plus 2 standard deviations. Analysis of the single antigen ELISA results was performed as previously described [14,24]. Briefly, each serum sample was considered positive if the mean absorbance was greater than the cut-off point. For the multiple-antigen ELISA results, single antigen ELISA data was re-evaluated and the serum tested was determined to be positive according to the following criteria: (1) any 2 or more antigens specifically react with serum with the absorbance greater than the cut-off value calculated from the mean absorbance (A_405nm_) plus 2 standard deviations of healthy controls or (2) any antigen that specifically reacts with serum when the cut-off value was calculated from the mean absorbance (A_405nm_) plus 3 standard deviations of healthy controls [25].

### Statistical analysis

Statistical analysis on human sera reactivity was performed using the Mann Whitney test within the GraphPad Prism® version 4.0 (GraphPad Software) software package.

## Results

### Evaluation of recombinant proteins as serodiagnostic reagents

The 4 purified recombinant proteins, TssD-5, Omp3, Omp85 and smBpF4, were used in an ELISA to detect antigen-specific IgG in culture confirmed or IFAT confirmed melioidosis sera as well as in *B. pseudomallei*-negative disease control sera and healthy individuals (non-melioidosis).

The majority (70%) of sera samples from culture- or IFAT- confirmed individuals used in this study were from the Malay ethnic group (Table 2). The incidence of melioidosis in other ethnic groups such as Chinese and Indians (13% each) appeared to be less frequent compared to the Malay group. The male to female ratio was 4.42 to 1. The number of males outnumbering females is consistent with other studies on the epidemiology of melioidosis in Malaysia: Pahang state (3.6:1) [22] and Alor Setar, Kedah state (3.03:1) [26]. In addition, 62% of the sera samples were from patients aged between 30–70 years. The human sera were tested against *B. pseudomallei* lysate by ELISA prior to performing the screen with the recombinant proteins. All 68 melioidosis patients’ sera reacted with *B. pseudomallei* lysate whilst the non-melioidosis control sera showed no reactivity (data not shown), demonstrating the sensitivity of ELISA screening.

**Figure 1:**
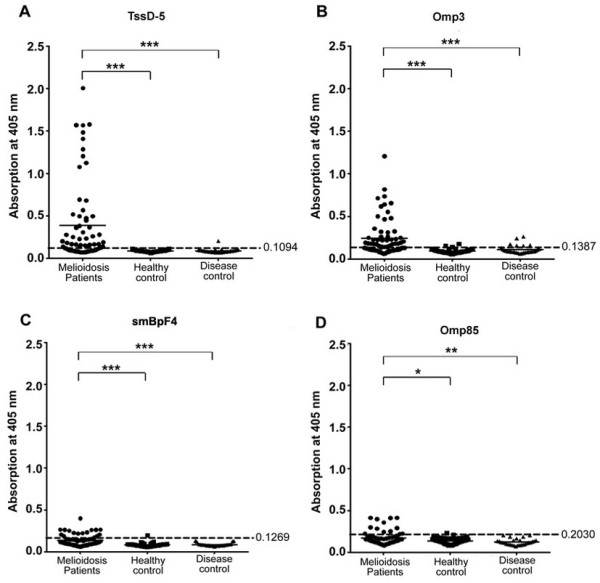
**Reactivity of human sera with 4 recombinant *B. pseudomallei *proteins.** Purified recombinant (**A**) TssD-5, (**B**) Omp3, (**C**) smBpF4 and (**D**) Omp85 were screened against melioidosis patients’ (n = 68, solid circle), healthy control (n = 61, solid square) and disease control (n = 29, solid triangle) sera. Each symbol represents the average of triplicate readings of one serum sample. Shown is a representative of 2 independent experiments. Cut-off values indicated by dashed line. Significance determined using the Mann Whitney test (***P-value < 0.0001; **P-value < 0.001; *P-value < 0.01).

All 4 recombinant *B. pseudomallei* proteins demonstrated varying sensitivity but good specificity (Table 3 and Figure 1). In brief, TssD-5 when used as a single antigen demonstrated the highest sensitivity in both culture confirmed (62%) and IFAT confirmed (85%) samples with an average of 71% and good specificity (96%) with a cut-off value of 0.1094, the lowest amongst the 4 antigens. Omp3 demonstrated moderate sensitivity of 50% in culture confirmed samples and 73% in IFAT confirmed samples with average of 59% with good specificity (90%) and the cut-off value was 0.1387. smBpF4 also demonstrated moderate but slightly lower sensitivity of 41% on average (38% in culture confirmed and 46% in IFTA confirmed samples, respectively) with good specificity (89%) and its cut-off value was 0.1269. Omp85 demonstrated the lowest sensitivity (19%) in both culture and IFTA confirmed samples but good specificity (96%) with its cut-off value calculated at 0.203. When all the 4 antigens were combined at equal ratios, this cocktail formulation demonstrated somewhat lower sensitivity of 65% on average (64% in culture confirmed and 65% in IFAT confirmed samples, respectively) compared to TssD-5 alone but improved specificity (99%). To predict the potential value of combining these 4 antigens under optimal conditions, we used 2 different criteria (Table 3) [25] to analyse the individual reactivity of TssD-5, Omp3, smBpF4 and Omp85 with the sera. By adopting these criteria, the detection sensitivity improved to 88.2% without compromising the specificity (96%). Great variation in antibody binding towards the same antigen was observed and this was particularly obvious for TssD-5 where the absorption values of individual sera ranged from 0.11 to 2.0 and to the lesser extent against Omp3 (0.145-1.2). The mean absorbance of all 4 recombinant proteins was significantly higher in melioidosis patients sera compared to non-melioidosis sera (Figure 1).

**Table 3:** ELISA results for melioidosis confirmed and control sera

**Recombinant proteins**	**Cut-off**	Sensitivity (%) (no. positive/total no.*)			**Specificity (%) (no. positive/total no. †)**
		**Culture confirmed**	**IFAT confirmed**	**Combined**	
		**(n = 42)**	**(n = 26)**	**(n = 68)**	
TssD-5 (type VI secretion system HCP protein)	0.1094	62 (26/42)	85 (22/26)	71 (48/68)	96 (4/90)
Omp3 (outer membrane protein A)	0.1387	50 (21/42)	73 (19/26)	59 (40/68)	90 (9/90)
smBpF4 (serine protease MprA)	0.1269	38 (16/42)	46 (12/26)	41 (28/68)	89 (10/90)
Omp85 (outer membrane protein)	0.2030	19 (8/42)	19 (5/26)	19 (13/68)	96 (4/90)
Cocktails of 4 recombinant proteins^§^	0.1936	64 (27/42)	65 (17/26)	65 (44/68)	99 (1/82**)
Multiple antigens combination (all 4 recombinant proteins^‡^)				88.2 (60/68)	96 (4/90)

* melioidosis confirmed sera by ELISA against *B. pseudomallei* lysate.

† non-melioidosis sera [healthy control (n = 61) and disease control (n = 29)].

** non-melioidosis sera [healthy control (n = 59) and disease control (n = 23)].

^§^ Cocktail of 4 recombinant proteins used to coat a single well.

‡ The single antigen ELISA data was re-evaluated and different criteria were used to calculate sensitivity and specificity. The serum tested was determined to be melioidosis positive according to the following criteria: (1) any 2 or more antigens specifically react with serum with the absorbance greater than the cut-off value calculated from the mean absorbance (A_405nm_) plus 2 standard deviations of healthy controls or (2) any antigen that specifically reacts with serum when the cut-off value was calculated from the mean absorbance (A_405nm_) plus 3 standard deviations of healthy controls.

## Discussion

A recent study reported the true sensitivity of culture as a diagnostic tool as 60% [27]. Thus, there is critical need for a fast, easy and accurate diagnostic assay for melioidosis as well as to differentiate individuals with active infection from those previously exposed to the bacteria, particularly in endemic countries [28]. Recombinant proteins as diagnostic antigens offer many advantages over the use of bacterial cells including eliminating health risks to laboratory personnel and reducing inconsistent antigen preparation [28]. Therefore, the use of recombinant proteins as potential diagnostic antigens via an ELISA detection-based screen were evaluated but with varying results. Recombinant-truncated flagellin was tested by ELISA as a diagnostic antigen and demonstrated high sensitivity and specificity (93.8% and 96.3%, respectively) [14], but only a limited number of samples from non-endemic regions was used. BipB and BipD were also evaluated as antigens by screening samples from 2 endemic regions, Thailand and Australia. However, neither of them demonstrated better diagnostic accuracy than the IHA [16].

In this study, TssD-5 demonstrated the highest sensitivity (71%) and good specificity (96%) within the cohort of sera samples. Furthermore, TssD-5 demonstrated minimal antibody response in healthy controls as compared to the other 3 antigens with only one weakly positive response observed in disease controls. This species-specific feature is valuable in endemic regions where high sero-positivity in healthy individuals poses a challenge to accurate diagnosis of melioidosis [29]. Omp3, on the other hand, demonstrated only moderate sensitivity (59%) although an earlier study on the same protein reported higher sensitivity (95%) [30], which may be attributed to different cut-off values used. In our study, control sera were obtained from a cohort of Malaysian individuals and therefore classified as from an endemic region. As such, it is not surprising that baseline sero-positivity (cut-off value) was high in the healthy population indicating possible previous exposure to *B. pseudomallei*. Of the previously reported immunogenic proteins, smBpF4 and Omp85 exhibited moderate to poor sensitivity of 41% and 19%, respectively, but showed good specificity (89-96%). Nonetheless, a PCR assay targeting the *mprA* gene of *B. pseudomallei* demonstrated 100% sensitivity and specificity in detecting the gene in clinical samples [31,32]. This suggests that whilst the *mprA* (smBpF4) gene may be a good bioindicator of *B. pseudomallei* by PCR, the protein may be unsuitable for serodiagnosis of melioidosis.

The evaluation of a cocktail of 4 proteins gave an improvement in specificity (99%) but the sensitivity (65%) was somewhat lower than TssD-5 alone (Table 3 and Figure 1). This finding was unexpected as the combination of antigens was predicted to improve the sensitivity or be equally sensitive as TssD-5. A similar finding was reported by Lyashchenko et al. [33] where combinatorial cocktails of 4 or 8 antigens resulted in significant loss of sensitivity for detection of *Mycobacterium tuberculosis* compared to the use of a single antigen. This reduced sensitivity may be explained by the limited protein binding capacity of the polystyrene solid phase in the microtitre plate, resulting in the reduced display of epitopes or proteins competing with each other for binding to polystyrene which may result in larger proteins swathing their smaller counterparts [33].

Zhang et al. [25] demonstrated that the heterogeneous antibody response to different antigens could be utilised to increase the sensitivity of diagnosis. This laid the foundation for the development of a multiple-antigen ELISA system for screening of tuberculosis with improved sensitivity and specificity over conventional diagnostic methods [25]. We used a similar approach by combining the 4 antigens, to achieve an improved sensitivity from 71% using a single antigen to 88.2% whilst retaining good specificity (96%).

Using a *B. pseudomallei* protein microarray, Felgner et al. [34] identified 49 potential serodiagnostic antigens for melioidosis including Omp3 confirming the potential of Omp3 as a candidate antigen. However, to date, none of these antigens have been proposed as a sensitive or specific diagnostic tool for melioidosis. A large number of these serodiagnostic proteins were classified as extracellular or outer membrane proteins as predicted by the computational annotation PSORTb and most of them are encoded on Chromosome 2 of *B. pseudomallei* K96243. TssD-5 satisfies both criteria and in addition, the up-regulation of the TssD-5 gene transcript during the bacteria’s adaptation to the intracellular environment proposes that TssD-5 is a potentially better biomarker candidate for diagnosis of melioidosis. Furthermore, our study suggests that protein size may be an important factor to consider when identifying sensitive sero-diagnostic antigens. TssD-5 (22.9 kDa) and Omp3 (27 kDa) demonstrated better diagnostic values compared to the larger proteins, smBpF4 (55 kDa) and Omp85 (85 kDa). Indeed, among 49 potential diagnostic proteins for melioidosis reported by Felger et al., more than a half were < 60 kDa [34]. Smaller molecular weight proteins have also been reported as sensitive diagnostic antigens for tuberculosis [25], acute leptospirosis [35] and *Brucella abortus* infection [36]. In summary, the present study demonstrated that TssD-5 is a potentially superior diagnostic candidate for melioidosis.

## Conclusions

We screened 4 recombinant *B. pseudomallei* proteins as potential diagnostic markers for melioidosis in an ELISA system. The recombinant Type VI secretion system protein TssD-5, achieved 71% sensitivity and 96% specificity, which is superior to the culture method with a recently reported sensitivity of 60%. The use of a multiple-antigen ELISA demonstrated better sensitivity (88.2%) while retaining good specificity (96%). Thus, TssD-5 in combination with the other 3 recombinant proteins tested demonstrated good potential as safe and sensitive serodiagnostic agents for melioidosis.

## Competing interests

The authors declare that they have no competing interests.

## Authors’ contributions

YH, CYC and SN conceived and designed the experiments. YH and CYC performed the experiments: RM and SDP provided reagents: YH, CYC and SN analysed the data: YH, CYC and SN wrote the manuscript. SDP critically reviewed the manuscript. All authors read and approved the final version of the manuscript.

## Pre-publication history

The pre-publication history for this paper can be accessed here:

http://www.biomedcentral.com/1471-2334/13/165/prepub
